# *Mycoplasma pneumoniae* Infections in Hospitalized Children — United States, 2018–2024

**DOI:** 10.15585/mmwr.mm7423a1

**Published:** 2025-06-26

**Authors:** Maureen H. Diaz, Adam L. Hersh, Jared Olson, Samir S. Shah, Matt Hall, Chris Edens

**Affiliations:** ^1^Division of Bacterial Diseases, National Center for Immunization and Respiratory Diseases, CDC; ^2^Division of Infectious Diseases, Department of Pediatrics, University of Utah, Salt Lake City, Utah; ^3^Department of Pharmacy, Primary Children’s Hospital, Salt Lake City, Utah; ^4^Cincinnati Children’s Hospital Medical Center, Cincinnati, Ohio; ^5^Children’s Hospital Association, Lenexa, Kansas.

**Summary**

**What is already known about this topic?**

*Mycoplasma pneumoniae* is a common cause of community-acquired pneumonia (CAP) in school-aged children. In the United States, *M. pneumoniae* infection prevalence decreased during the COVID-19 pandemic and remained low through 2023.

**What is added by this report?**

The number of hospital discharges of children with *M. pneumoniae*–associated CAP from U.S. pediatric hospitals increased sharply in 2024, accounting for approximately one half of hospitalized children with CAP. This number included children aged <5 years, a group in which *M. pneumoniae* infections have historically been less commonly reported. Data on length of hospitalization and intensive care unit admissions indicate that *M. pneumoniae* infections in 2024 were not more severe than 2018–2023 infections.

**What are the implications for public health practice?**

Increased awareness among health care providers might improve diagnosis and could guide treatment of *M. pneumoniae* infections among children of all ages, especially during periodic increases in *M. pneumoniae* circulation and among children requiring hospitalization.

## Abstract

*Mycoplasma pneumoniae* is a common bacterial cause of respiratory infection and a leading cause of childhood community-acquired pneumonia (CAP). Increases in *M. pneumoniae* infection occur every 3–5 years. In the United States, *M. pneumoniae* prevalence decreased during and immediately after the COVID-19 pandemic. Information from 42 U.S. children’s hospitals that provided information to the Pediatric Health Information System, a database of clinical and resource use information, was used to identify discharge diagnostic codes for 2018–2024 indicating *M. pneumoniae* infection. *M. pneumoniae*–associated CAP incidence among children aged ≤18 years was significantly higher in 2024 (12.5 per 1,000 hospitalizations) than during 2018–2023 (2.1). During the study period, an *M. pneumoniae* diagnostic code was listed in 11.5% of pediatric CAP hospitalizations, peaking at 53.8% in July 2024. Among pediatric *M. pneumoniae* CAP cases, the highest percentage occurred among children aged 6–12 years (42.6%), followed by children aged 2–5 years (25.7%) and 13–18 years (21.1%). The lowest occurred among those aged 12–23 months (6.4%) and 0–11 months (4.2%). *M. pneumoniae* infections in 2024 were not more severe than 2018–2023 infections, as assessed by length of hospitalization and percentage of patients admitted to an intensive care unit. The increase in *M. pneumoniae* infections in the United States in 2024 might be higher than previous periodic increases because the susceptible population was larger after sustained low incidence during and immediately after the COVID-19 pandemic. Health care providers should be aware of the periodicity of *M. pneumoniae* CAP and consider testing for this pathogen as a cause of respiratory illness among children of all ages.

## Introduction

*Mycoplasma pneumoniae* is a common cause of bacterial respiratory infections, including community-acquired pneumonia (CAP). Most *M. pneumoniae* infections are mild, although some patients develop pneumonia requiring hospitalization (*1*). *M. pneumoniae* infections affect all age groups; however, the highest percentages of cases have historically been reported among children and adolescents aged 5–17 years. Previous studies have estimated that *M. pneumoniae* accounts for approximately 10%–30% of hospitalized pediatric CAP cases (*1*,*2*). No vaccine is available to prevent *M. pneumoniae* infection. Macrolide antibiotics such as azithromycin, clarithromycin, and erythromycin are the first-line treatment for infection.* Macrolide-resistant *M. pneumoniae* infections are widespread in some regions of the world but remain relatively uncommon in the United States, accounting for <10% of cases (*3*,*4*).

Historically, *M. pneumoniae* infections have increased approximately every 3–5 years, which mathematical modeling suggests is due, in part, to changes in predominant strain types and associated increases in susceptible populations resulting from waning immunity after infection (*1*,*5*). During the COVID-19 pandemic, *M. pneumoniae* infections were rarely detected (*6*). In 2023, *M. pneumoniae* infections increased in other countries but remained low in the United States (*7*). *M. pneumoniae* infections in the United States began to increase sharply in April 2024, as indicated by an increase in the percentage of positive test results and syndromic surveillance data from emergency departments.^†^ This report describes the epidemiology of *M. pneumoniae* and characterizes infections among patients aged ≤18 years (referred to as children in this report) discharged from pediatric hospitals during 2024 compared with previous years.

## Methods

### Population and Data Source

The Pediatric Health Information System (PHIS)^§^ contains clinical and resource use data for patients aged ≤18 years. Children treated at one of 42 U.S. children’s hospitals that consistently contributed data to PHIS were eligible for inclusion. The PHIS database was queried for *International Classification of Diseases, Tenth Revision* (ICD-10) discharge diagnostic codes indicating CAP^¶^ and *M. pneumoniae* infection.** Data were used to identify the total number of CAP cases, *M. pneumoniae*–associated CAP cases, and CAP cases with administration of an antimicrobial agent effective against *M. pneumoniae*^††^ during January 2018–December 2024. ICD-10 codes used to identify *M. pneumoniae* CAP were validated by comparing discharge diagnosis codes with laboratory results at one hospital (Primary Children’s Hospital, Salt Lake City, Utah).

### Analysis

To determine whether the severity of *M. pneumoniae* infections during 2018–2023 (before, during, and immediately after the COVID-19 pandemic) differed from the severity of infections in 2024, as measured by intensive care unit admission and length of hospital stay, the number and rate (cases per 1,000 hospitalizations) of *M. pneumoniae* cases during 2018–2024, 2018–2023, and 2024 were analyzed. The number and percentage of cases during each period were reported by age group, sex, race and ethnicity, and characteristics of hospitalization. Chi-square and Wilcoxon rank-sum tests were used to compare demographic and clinical characteristics of patients with infections during 2018–2023 and 2024, with p-values <0.05 considered statistically significant. Statistical testing was performed using SAS (version 9.4; SAS Institute). This activity was reviewed by CDC, deemed not research, and was conducted consistent with applicable federal law and CDC policy.^§§^

## Results

### Prevalence of CAP-Associated Pediatric Hospitalizations

**FIGURE 1:**
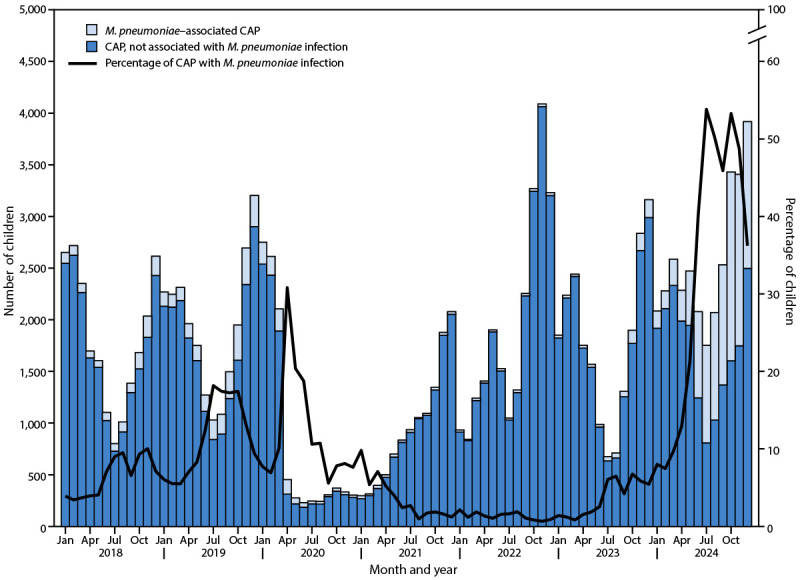
Hospitalized children with community-acquired pneumonia, associated and not associated* with *Mycoplasma pneumoniae*, by month — Pediatric Hospital Information System,^†^ United States, 2018–2024 **Abbreviation: **CAP = community-acquired pneumonia. * The number of CAP cases that were not associated with *M. pneumoniae* infection was calculated by subtracting the number of *M. pneumoniae* CAP cases from the total number of CAP cases. ^†^ Forty-two hospitals.

Among 5,631,734 hospitalized children, 141,955 (2.5%) received a CAP diagnosis (Figure 1), including 111,064 (2.3%) of 4,760,521 during 2018–2023 and 30,891 (3.5%) of 871,213 in 2024. Seasonal increases in CAP occurred annually during the fall and winter, except during 2020–2021. The annual number of CAP cases ranged from 10,221 in 2020 to 30,891 in 2024.

### Percentage of Pediatric CAP Cases with an *M. pneumoniae* Diagnostic Code

Overall, among all hospitalized pediatric patients with CAP, an *M. pneumoniae* diagnostic code was listed for 16,353 of 141,955 (11.5%); 94.4% of identified *M. pneumoniae* infections had a CAP diagnosis. *M. pneumoniae* accounted for <5% of total hospitalized CAP cases annually during 2021–2023, then increased to 33% in 2024, peaking at 53.8% in July 2024 (Figure 1). Among 16,353 *M. pneumoniae*–related hospital discharges (representing 4.45 per 1,000 hospitalizations), a total of 6,055 (2.12 per 1,000) occurred during 2018–2023, and 10,298 (12.49 per 1,000) occurred in 2024 (Table).

**TABLE:** Demographic and clinical characteristics of children hospitalized with *Mycoplasma pneumoniae–*associated community-acquired pneumonia — Pediatric Hospital Information System, United States, 2018–2024

Characteristic	2018–2024	2018–2023	2024	p-value^†^
	Rate* (95% CI): 4.45 (4.38–4.52)	Rate* (95% CI): 2.12 (2.07–2.18)	Rate* (95% CI): 12.49 (12.26–12.74)	
	No. (%)	No. (%)	No. (%)	
**Total**	**16,353 (100)**	**6,055 (100)**	**10,298 (100)**	**<0.001**
**Age group, yrs**				
<1	690 (4.2)	285 (4.7)	405 (3.9)	<0.001
1	1,046 (6.4)	384 (6.3)	662 (6.4)	
2–5	4,210 (25.7)	1,491 (24.6)	2,719 (26.4)	
6–12	6,959 (42.6)	2,474 (40.9)	4,485 (43.6)	
13–18	3,448 (21.1)	1,421 (23.5)	2,027 (19.7)	
**Sex**				
Female	7,192 (44.0)	2,726 (45.0)	4,466^§^ (43.4)	0.07
Male	9,159 (56.0)	3,329 (55.0)	5,830^§^ (56.6)	
**Race and ethnicity**				
Asian, non-Hispanic	843 (5.2)	308 (5.1)	535 (5.2)	<0.001
Black or African American, non-Hispanic	2,027 (12.4)	750 (12.4)	1,277 (12.4)	
Hispanic or Latino	4,192 (25.6)	1,553 (25.6)	2,639 (25.6)	
White, non-Hispanic	8,223 (50.3)	2,959 (48.9)	5,264 (51.1)	
Other	1,068 (6.5)	485 (8.0)	583 (5.7)	
**Clinical characteristics and outcomes **				
Length of hospitalization stay, median (IQR)	2 days (1–4 days)	3 days (2–6 days)	2 days (1–4 days)	<0.001
CAP diagnosis^¶^	15,440 (94.4)	5,549 (91.6)	9,891 (96.0)	<0.001
Admitted to intensive care unit	3,586 (21.9)	1,577 (26.0)	2,009 (19.5)	<0.001
Received antibiotics for M. pneumoniae**	15,682 (95.9)	5,774 (95.4)	9,908 (96.2)	0.008
Died^††^	44 (0.3)	29 (0.5)	15 (0.1)	<0.001

**Abbreviation:** CAP = community-acquired pneumonia.

* Number of cases per 1,000 hospitalizations.

^†^ Chi-square (for categorical variables) and Wilcoxon rank-sum (for continuous variables) tests were used to compare demographic and clinical characteristics of patients with infections during 2018–2023 and 2024.

^§^ Missing values (2) in 2024.

^¶^ Influenza due to identified novel influenza A virus with pneumonia (J09.X1); influenza due to other identified influenza virus with pneumonia (J10.00–10.01 and J10.08); influenza due to unidentified influenza virus with pneumonia (J11.00 and J11.08); viral pneumonia, not elsewhere classified (J12.0–12.3 and J12.8–12.9); pneumonia due to *Streptococcus pneumoniae* (J13); pneumonia due to *Haemophilus influenzae* (J14); bacterial pneumonia, not elsewhere classified (J15.0–15.9); pneumonia due to other infectious organisms, not elsewhere classified (J16.0 and J16.8); pneumonia in diseases classified elsewhere (J78); bronchopneumonia, unspecified organism (J18.0–18.2 and J18.8–18.9); acute respiratory distress syndrome (J80); Legionnaires disease (A48.1); and acute bronchitis due to *M. pneumoniae* (J20.0).

**Antibiotics considered effective against *M. pneumoniae*, with *Current Procedural Terminology* codes and exclusions, include azithromycin (122421, excluding ophthalmic 1224214568000 and 1224214568064); clarithromycin (122425); doxycycline (123115, excluding topical 1231154041000,1231154042000, and 1231154045652); minocycline (123131); levofloxacin (123215, excluding inhalation 1232154242272 and excluding ophthalmic 1232154565000, 1232154565064, 1232154565114, and 1232154569000); and moxifloxacin (123225, excluding ophthalmic 1232254539000, 1232254539591, 1232254539861, 1232254539941, 1232254565000, 1232254565074, and 1232254565114).

^††^ The median age of children with *M. pneumoniae* CAP who died was 12.0 years (IQR: 2.0–16.5 years).

### Demographic and Clinical Characteristics of Children Hospitalized with *M. pneumoniae* and CAP

**FIGURE 2:**
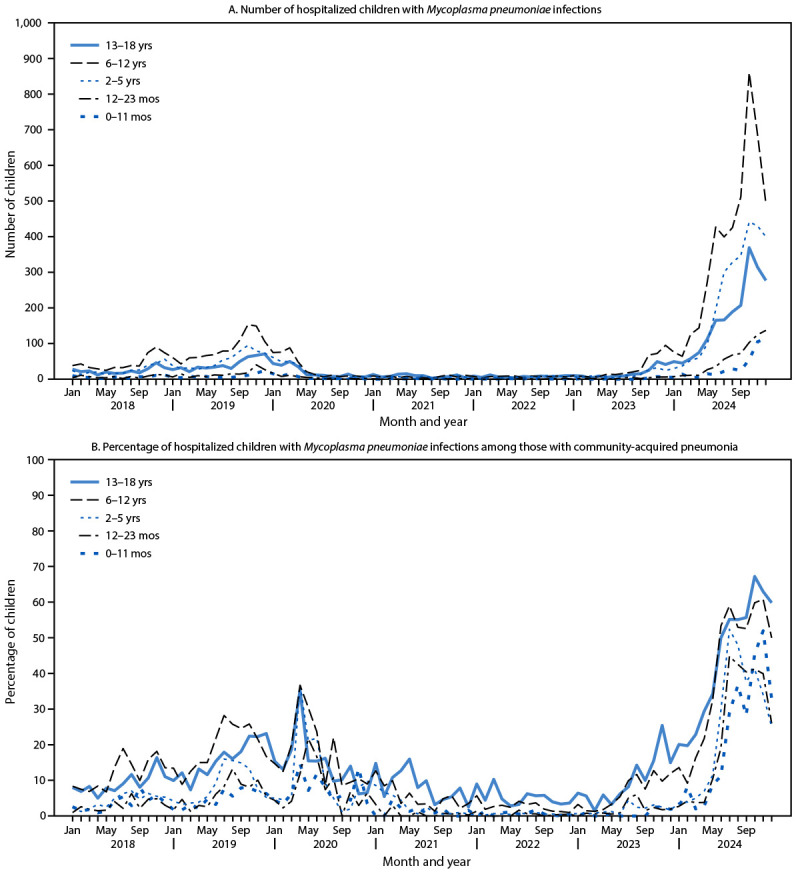
Number of hospitalized children with *Mycoplasma pneumoniae* infections (A) and percentage of children with *M. pneumoniae* infections among those with community-acquired pneumonia (B), by month and age group — Pediatric Hospital Information System,* United States, 2018–2024 * Forty-two hospitals.

The number of hospital discharges for *M. pneumoniae*–associated CAP decreased in early 2020, remained low through 2023, and increased in all age groups in 2024 (Figure 2). The highest total number and proportion of *M. pneumoniae* CAP cases occurred among children aged 6–12 years (6,959; 42.6%), followed by those aged 2–5 years (4,210; 25.7%) and 13–18 years (3,448; 21.1%); the lowest proportion was among children aged 12–23 months (1,046; 6.4%) and 0–11 months (690; 4.2%) (Table). The peak monthly proportion of CAP cases attributed to *M. pneumoniae* was highest among children aged 13–18 years (67.2%), followed by those aged 6–12 years (60.8%), 2–5 years (53.4%), 0–11 months (52.0%), and 12–23 months (44.8%) (Figure 2). In 2024, compared with 2018–2023, the proportion of CAP attributed to *M. pneumoniae* increased the most among children aged 12–23 months (increased 8.5 times), followed by 0–11 months (8.1 times), 2–5 years (7.7 times), 13–18 years (4.5 times), and 6–12 years (4.1 times). Compared with 2018–2023, the length of hospital stay in 2024 was shorter (2 days [range: 1–4 days] versus 3 days [range: 2–6 days]), and the percentage of patients admitted to an intensive care unit was lower (19.5% versus 26.0%). Forty-four (0.3%) deaths occurred among children with *M. pneumoniae* CAP, including 29 (0.5% of *M. pneumoniae* CAP cases) during 2018–2023 and 15 (0.1%) in 2024. The median age of patients who died from *M. pneumoniae* CAP was 12 years (IQR: 2.0–16.5 years).

ICD-10 codes used for identifying *M. pneumoniae* CAP were validated by comparing discharge diagnosis codes with laboratory results from one hospital (Primary Children’s Hospital, Salt Lake City, Utah) for 2018–2024. A positive polymerase chain reaction test result for *M. pneumoniae* was recorded for 86% of discharges coded as *M. pneumoniae* pneumonia; 14% of cases did not have an *M. pneumoniae*–specific test result code recorded. Code J15.7 (pneumonia due to *Mycoplasma pneumoniae*) was recorded for 84% of discharges coded as *M. pneumoniae* CAP. During the study period in all 42 hospitals, 22.0% of all CAP inpatients and 95.9% of *M. pneumoniae* CAP inpatients received an antibiotic typically considered effective against *M. pneumoniae* (i.e., a macrolide antibiotic); the proportion of *M. pneumoniae* CAP patients who received these antibiotics was slightly higher in 2024 (96.2%) than during 2018–2023 (95.4%) (Table).

## Discussion

Consistent with recently reported trends worldwide (*6*,*7*), analyses of data from 42 U.S. children’s hospitals indicate that discharges for *M. pneumoniae* CAP decreased in early 2020, remained low through 2023, and increased in all age groups in 2024. During July–December 2024, *M. pneumoniae* ICD-10 codes were listed for approximately one half of CAP hospitalizations at U.S. children’s hospitals, the highest level in 6 years. Increases in *M. pneumoniae* CAP were not observed during annual seasonal increases in overall CAP during 2021–2023.

Increases in *M. pneumoniae* infection occur approximately every 3–5 years, likely due to variations in strain predominance (*5*). The 2024 increase in the United States and other countries was higher than most previously reported periodic increases (*1*,*2*). Surveillance data and mathematical modeling suggest that this increase might reflect increased population susceptibility after low levels of *M. pneumoniae* circulated worldwide during and immediately after the COVID-19 pandemic (*7*–*9*). Despite the increased population susceptibility, pediatric *M. pneumoniae* infections requiring hospitalization in 2024 did not appear to be more severe than those during the previous 5 years.

Historically, the highest percentage of *M. pneumoniae* infections have been reported among children aged 5–17 years (*1*,*10*). In this study, children aged 6–12 years similarly accounted for the highest number and percentage of *M. pneumoniae* CAP cases. However, comparing 2024 with 2018–2023, the proportion of CAP attributed to *M. pneumoniae* increased the most among children aged <5 years. In addition, although the number of *M. pneumoniae* infections among children aged <2 years was lower than that in older children and adolescents, *M. pneumoniae* accounted for approximately one half of CAP among children aged 0–11 months and 12–23 months at peaks in November and July 2024, respectively.

These findings suggest that during periodic increases in *M. pneumoniae* infections, this pathogen might account for a substantial proportion of CAP among children of all ages, including those aged <5 years. Widespread use of multiplex laboratory tests for detection of respiratory pathogens could contribute to improved recognition of infections, including *M. pneumoniae* infections, in younger patients. The high percentage of patients with an *M. pneumoniae*–associated ICD-10 code with confirmatory laboratory evidence at one reporting site suggests that discharge code data at children’s hospitals can be used to accurately track infection trends over time.

Health care providers should be aware of increases in *M. pneumoniae* CAP, which might occur in summer and fall when circulation of other common respiratory pathogens is low (*1*). Because *M. pneumoniae* infection cannot be identified based on physical examination alone, providers should consider and test for this pathogen as a cause of respiratory illness among children of all ages, especially during periods of high transmission. Confirmation of *M. pneumoniae* infection by laboratory testing helps guide patient treatment because first-line antibiotic treatment of *M. pneumoniae* CAP differs from that for CAP of other bacterial etiologies.

### Limitations

The findings in this report are subject to at least four limitations. First, passively collected resource use data are subject to possible biases from test ordering and medical coding practices. A limited evaluation at a single hospital indicated that most *M. pneumoniae*–associated discharges were supported by laboratory testing; however, this might not be generalizable to all facilities and might result in an underestimation of cases. Second, coinfections and underlying conditions were not evaluated, which might affect comparison of clinical characteristics between study periods. Third, laboratory results for characterization of *M. pneumoniae*, including antimicrobial susceptibility testing, were not available for cases included in this analysis, which could also affect study period comparisons. Finally, because most *M. pneumoniae* infections are mild, cases requiring hospitalization likely accounted for a small proportion of infections during the study period.

### Implications for Public Health Practice

Increased awareness among health care providers might improve diagnosis and could guide treatment of *M. pneumoniae* infections among children of all ages, especially during periodic increases in *M. pneumoniae* circulation and among children requiring hospitalization. In addition, ongoing surveillance of *M. pneumoniae* infections is important to detect periodic increases and improve mathematical modeling to predict the timing and magnitude of future increases. Characterization of circulating *M. pneumoniae* strains is needed to monitor predominant genotypes, emergence of variants, and antimicrobial resistance patterns.

## Footnotes

*
CDC | Clinical care of *Mycoplasma pneumoniae* infection

†
CDC | *Mycoplasma pneumoniae* infections have been increasing

§
Children’s Hospital Association | Leverage your data with CHA’s Pediatric Health Information System

¶ Influenza due to identified novel influenza A virus with pneumonia (J09.X1); influenza due to other identified influenza virus with pneumonia (J10.00–10.01 and J10.08); influenza due to unidentified influenza virus with pneumonia (J11.00 and J11.08); viral pneumonia, not elsewhere classified (J12.0–12.3 and J12.8–12.9); pneumonia due to *Streptococcus pneumoniae* (J13); pneumonia due to *Haemophilus influenzae* (J14); bacterial pneumonia, not elsewhere classified (J15.0–15.9); pneumonia due to other infectious organisms, not elsewhere classified (J16.0 and J16.8); pneumonia in diseases classified elsewhere (J78); bronchopneumonia, unspecified organism (J18.0–18.2 and J18.8–18.9); acute respiratory distress syndrome (J80); Legionnaires disease (A48.1); and acute bronchitis due to *M. pneumoniae* (J20.0).

** Pneumonia due to *M. pneumoniae* (J15.7); acute bronchitis due to *M. pneumoniae* (J200); *Mycoplasma* infection, unspecified site (A493); and *M. pneumoniae* as the cause of diseases classified elsewhere (B960).

†† Antibiotics considered effective against *M. pneumoniae*, with *Current Procedural Terminology* codes and exclusions, include azithromycin (122421, excluding ophthalmic 1224214568000 and 1224214568064); clarithromycin (122425); doxycycline (123115, excluding topical 1231154041000, 1231154042000, and 1231154045652); minocycline (123131); levofloxacin (123215, excluding inhalation 1232154242272 and excluding ophthalmic 1232154565000, 1232154565064, 1232154565114, and 1232154569000); and moxifloxacin (123225, excluding ophthalmic 1232254539000, 1232254539591, 1232254539861, 1232254539941, 1232254565000, 1232254565074, and 1232254565114).

§§ 45 C.F.R. part 46.102(l)(2), 21 C.F.R. part 56; 42 U.S.C. Sect. 241(d); 5 U.S.C. Sect. 552a; 44 U.S.C. Sect. 3501 et seq.
